# Supplementary material for: Modeling glioblastoma heterogeneity as a dynamic network of cell states
Source: Mol Syst Biol. 2021 Sep 16;17(9):e10105. doi: 10.15252/msb.202010105 (PMC8444284; doi:10.15252/msb.202010105)
Supplement: Supplementary file 5 — Source Data for Figure 3 [file MSB-17-e10105-s001.zip › Figure3A_sourcedata/GSEA_3065/hallmarks_state6.GseaPreranked.1623416308702/HALLMARK_ANDROGEN_RESPONSE.html]

Details for gene set HALLMARK\_ANDROGEN\_RESPONSE[GSEA]

|  || Dataset | state6 |
| Phenotype | NoPhenotypeAvailable |
| Upregulated in class | na\_neg |
| GeneSet | HALLMARK\_ANDROGEN\_RESPONSE |
| Enrichment Score (ES) | -0.40608197 |
| Normalized Enrichment Score (NES) | -1.5143977 |
| Nominal p-value | 0.011589404 |
| FDR q-value | 0.03629449 |
| FWER p-Value | 0.351 |
Table: GSEA Results Summary

  

Fig 1: Enrichment plot: HALLMARK\_ANDROGEN\_RESPONSE      
 Profile of the Running ES Score & Positions of GeneSet Members on the Rank Ordered List

  

| PROBE | GENE SYMBOL | GENE\_TITLE | RANK IN GENE LIST | RANK METRIC SCORE | RUNNING ES | CORE ENRICHMENT || 1 | RAB4A |  |  | 277 | 0.156 | -0.0009 | No |
| 2 | TSC22D1 |  |  | 398 | 0.136 | 0.0123 | No |
| 3 | ARID5B |  |  | 638 | 0.111 | 0.0069 | No |
| 4 | SAT1 |  |  | 744 | 0.102 | 0.0151 | No |
| 5 | CDK6 |  |  | 807 | 0.097 | 0.0273 | No |
| 6 | SMS |  |  | 1264 | 0.073 | -0.0105 | No |
| 7 | CCND1 |  |  | 1317 | 0.071 | -0.0025 | No |
| 8 | NDRG1 |  |  | 1488 | 0.065 | -0.0092 | No |
| 9 | ZBTB10 |  |  | 1502 | 0.064 | 0.0020 | No |
| 10 | DBI |  |  | 1549 | 0.062 | 0.0090 | No |
| 11 | APPBP2 |  |  | 1737 | 0.055 | -0.0014 | No |
| 12 | TPD52 |  |  | 1899 | 0.051 | -0.0099 | No |
| 13 | ANKH |  |  | 2098 | 0.045 | -0.0237 | No |
| 14 | XRCC5 |  |  | 2102 | 0.045 | -0.0152 | No |
| 15 | ZMIZ1 |  |  | 2143 | 0.043 | -0.0111 | No |
| 16 | NCOA4 |  |  | 2207 | 0.041 | -0.0102 | No |
| 17 | UAP1 |  |  | 2254 | 0.040 | -0.0076 | No |
| 18 | PMEPA1 |  |  | 2388 | 0.036 | -0.0156 | No |
| 19 | UBE2I |  |  | 2399 | 0.036 | -0.0096 | No |
| 20 | VAPA |  |  | 3021 | 0.022 | -0.0765 | No |
| 21 | PGM3 |  |  | 3268 | 0.016 | -0.1015 | No |
| 22 | ACTN1 |  |  | 3411 | 0.013 | -0.1153 | No |
| 23 | GSR |  |  | 3436 | 0.012 | -0.1156 | No |
| 24 | FKBP5 |  |  | 3790 | 0.005 | -0.1551 | No |
| 25 | CAMKK2 |  |  | 3991 | 0.001 | -0.1778 | No |
| 26 | CCND3 |  |  | 4016 | 0.000 | -0.1805 | No |
| 27 | RPS6KA3 |  |  | 4022 | 0.000 | -0.1810 | No |
| 28 | PIAS1 |  |  | 4383 | -0.007 | -0.2210 | No |
| 29 | ELK4 |  |  | 4672 | -0.012 | -0.2516 | No |
| 30 | ITGAV |  |  | 4722 | -0.013 | -0.2547 | No |
| 31 | ABCC4 |  |  | 5010 | -0.018 | -0.2839 | No |
| 32 | IQGAP2 |  |  | 5055 | -0.019 | -0.2851 | No |
| 33 | AKT1 |  |  | 5071 | -0.020 | -0.2829 | No |
| 34 | MYL12A |  |  | 5109 | -0.021 | -0.2830 | No |
| 35 | STK39 |  |  | 5381 | -0.026 | -0.3089 | No |
| 36 | HMGCS1 |  |  | 5446 | -0.027 | -0.3109 | No |
| 37 | B4GALT1 |  |  | 5870 | -0.036 | -0.3523 | No |
| 38 | DNAJB9 |  |  | 5974 | -0.037 | -0.3567 | No |
| 39 | HOMER2 |  |  | 6148 | -0.041 | -0.3683 | No |
| 40 | CENPN |  |  | 6194 | -0.042 | -0.3651 | No |
| 41 | XRCC6 |  |  | 6553 | -0.052 | -0.3959 | Yes |
| 42 | CDC14B |  |  | 6571 | -0.052 | -0.3875 | Yes |
| 43 | PDLIM5 |  |  | 6585 | -0.052 | -0.3786 | Yes |
| 44 | SEC24D |  |  | 6631 | -0.054 | -0.3732 | Yes |
| 45 | NGLY1 |  |  | 6841 | -0.060 | -0.3853 | Yes |
| 46 | SLC38A2 |  |  | 6934 | -0.063 | -0.3834 | Yes |
| 47 | UBE2J1 |  |  | 7018 | -0.065 | -0.3799 | Yes |
| 48 | LIFR |  |  | 7101 | -0.068 | -0.3759 | Yes |
| 49 | ELOVL5 |  |  | 7151 | -0.069 | -0.3678 | Yes |
| 50 | PA2G4 |  |  | 7192 | -0.071 | -0.3584 | Yes |
| 51 | SLC26A2 |  |  | 7256 | -0.073 | -0.3512 | Yes |
| 52 | PLPP1 |  |  | 7391 | -0.078 | -0.3512 | Yes |
| 53 | ABHD2 |  |  | 7643 | -0.088 | -0.3626 | Yes |
| 54 | INSIG1 |  |  | 7721 | -0.092 | -0.3532 | Yes |
| 55 | SRP19 |  |  | 7725 | -0.092 | -0.3352 | Yes |
| 56 | GNAI3 |  |  | 7760 | -0.093 | -0.3206 | Yes |
| 57 | IDI1 |  |  | 7851 | -0.099 | -0.3114 | Yes |
| 58 | SCD |  |  | 7908 | -0.102 | -0.2978 | Yes |
| 59 | ADRM1 |  |  | 7955 | -0.104 | -0.2824 | Yes |
| 60 | FADS1 |  |  | 8186 | -0.120 | -0.2850 | Yes |
| 61 | ACSL3 |  |  | 8295 | -0.132 | -0.2713 | Yes |
| 62 | DHCR24 |  |  | 8321 | -0.134 | -0.2475 | Yes |
| 63 | TMEM50A |  |  | 8329 | -0.136 | -0.2215 | Yes |
| 64 | AKAP12 |  |  | 8586 | -0.185 | -0.2143 | Yes |
| 65 | SPCS3 |  |  | 8633 | -0.204 | -0.1793 | Yes |
| 66 | B2M |  |  | 8718 | -0.258 | -0.1379 | Yes |
| 67 | HMGCR |  |  | 8759 | -0.301 | -0.0831 | Yes |
| 68 | LMAN1 |  |  | 8790 | -0.441 | 0.0007 | Yes |
Table: GSEA details [plain text format]

  

Fig 2: HALLMARK\_ANDROGEN\_RESPONSE: Random ES distribution      
 Gene set null distribution of ES for **HALLMARK\_ANDROGEN\_RESPONSE**
